# Supplementary material for: Room-temperature tetragonal non-collinear Heusler antiferromagnet Pt2MnGa
Source: Nat Commun. 2016 Aug 26;7:12671. doi: 10.1038/ncomms12671 (PMC5007462; doi:10.1038/ncomms12671)
Supplement: Supplementary Information — Supplementary Figures 1-2, Supplementary Notes 1-2 and Supplementary References [file ncomms12671-s1.pdf]

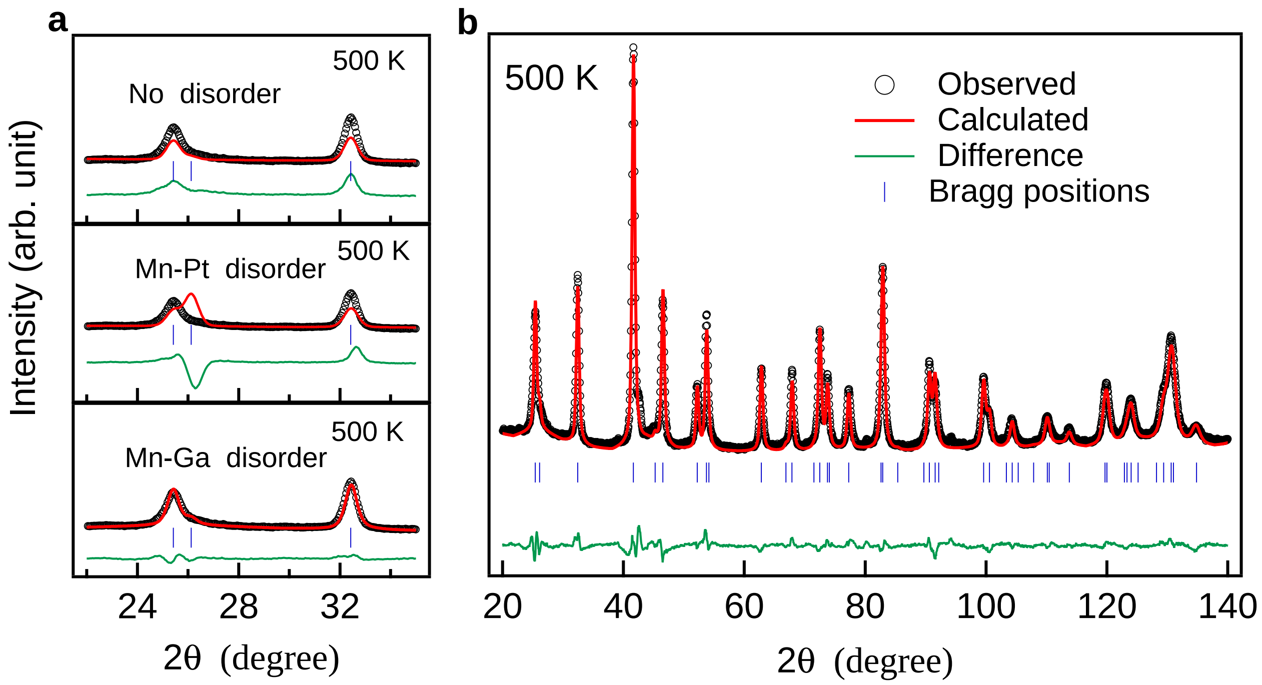

Supplementary Figure 1. Powder neutron diffraction on Pt_2MnGa at 500 K. The green curve shows the difference between observed (black) and calculated patterns (red). Vertical ticks indicate the nuclear Bragg peak positions. **a** The (002) and (110) Bragg peaks (black circles) have been fitted (red solid lines) by assuming (i) no disorder, (ii) Mn($2a$)/Pt($4d$) disorder, and (iii) Mn($2a$)/Ga($2b$) disorder. **b** Comparison of neutron diffraction patterns for the whole angular range.

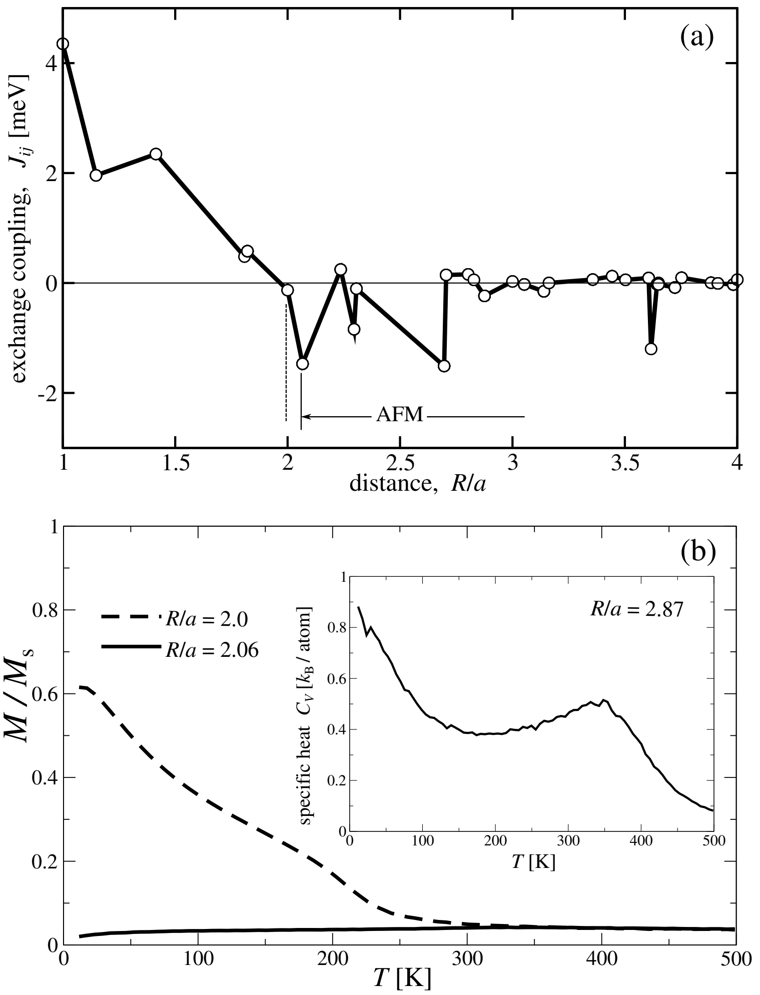

Supplementary Figure 2. a Isotropic exchange coupling constants J_{ij} computed as functions of distance R (in the units of a) between i and j sites within Mn($2a$) sublattice. **b** Monte-Carlo simulated $M/M_{\rm S}$ temperature dependencies of the Heisenberg model parametrized by the computed J_{ij}. Solid line corresponds to the minimal cluster size needed to set the AFM order, dashed - by one shall smaller cluster. The inset shows the temperature dependency of the specific heat $C_{\rm V}$ (computed for the largest technically possible cluster size of $R/a = 2.87$) which indicates the position of $T_{\rm N}$ by the local maximum.

Supplementary Note 1

Crystal structure from neutron diffraction

Supplementary Figure 1 shows the observed and calculated neutron diffraction patterns at 500 K (paramagnetic phase) in the range of $20^\circ < 2\theta < 140^\circ$. All peaks are well indexed in Rietveld refinement assuming the space group $I4/mmm$ as in case of the X-ray diffraction. Refined lattice parameters are $a = b = 4.03$ Å, $c = 7.24$ Å. In a perfectly ordered unit cell, Mn, Ga and Pt atoms would occupy $2a\,(0, 0, 0)$, $2b\,(0, 0, 0.5)$ and $4d\,(0, 0.5, 0.25)$ Wyckoff positions, respectively. On the other hand, our Rietveld fit indicates possible presence of Mn($2a$)-Ga($2b$) disorder: by comparing several reasonable chemical configurations (focusing on (002) and (110) Bragg peaks), the perfect agreement is provided by assuming $\sim 33\,\%$ of Mn($2a$)-Ga($2b$) disorder (lower panel in Supplementary Figure 1 a).

Supplementary Note 2

Monte-Carlo simulations

Here we try to figure which mechanisms are responsible for setting such a long range ground-state modulation (by assuming a perfectly ordered structure). Since the relativistic effects do not affect the ground-state $\mathbf{q}$ vector substantially, in the following we calculate the isotropic exchange coupling constants J_{ij} using the real-space approach[1] implemented in the SPR-KKR Green's function method.[2] In Supplementary Figure 2 a they are plotted as functions of distance between the interacting sites i and j. Here, we drop all interactions involving Pt and Ga atoms as insignificant,

by leaving only those between Mn atoms. It follows, that all nearest Mn$(2a)$-Mn$(2a)$ interactions are parallel ($J > 0$), whereas the antiparallel ones ($J < 0$) are encountered by starting from $R/a = 2$ (6-th shell within $(2a)$-sublattice). As it is shown by $M(T)/M_\mathrm{S}$ curves (Supplementary Figure 2 b) obtained by the Monte-Carlo simulation (ALPS package[3]) of the classical Heisenberg model ($H = -\sum_{i>j} J_{ij}\,\mathbf{e}_i \cdot \mathbf{e}_j$, where $\mathbf{e}_{i,j}$ are the unity vectors along the local magnetization directions on i and j sites), the AFM order sets in by including all interactions at least up to $R/a \approx 2.06$ (7-th shell); accounting of the higher shells does not affect the $M(T)$ behavior anymore. Such a superposition of the strong nearest parallel and the weaker long-range antiparallel exchange interactions typically allows for the long-range spin-spiral order. Its direction ($\mathbf{q} \parallel [001]$) follows from the symmetry reasons: the 7-th shell, critical for setting up the AFM order, contains 8 atoms at $\mathbf{R} = (\pm a, 0, \pm c)$ and $(0, \pm a, \pm c)$, situated above and below the ab-plane of the central atom. The corresponding Neel temperature can be estimated from the peak of the magnetic specific heat $C_\mathrm{V}(T)$ computed for the largest cluster size ($T_\mathrm{N} \approx 350\,\mathrm{K}$ at $R/a \approx 2.87$, see the inset in Supplementary Figure 2 b). This again reasonably agrees with experimental $M(T)$ slope change.

Supplementary References

1. Liechtenstein, A. I., Katsnelson, M. I., Antropov, V. P. & Gubanov, V. A. Local spin density functional approach to the theory of exchange interactions in ferromagnetic metals and alloys. *J. Magn. Magn. Materials* **67**, 65–74 (1987).

2. Ebert, H., Ködderitzsch, D. & Minár, J. Calculating condensed matter properties using the KKR-Green's function method - recent developments and applications. *Rep. Prog. Phys.* **74**, 096501 (2011).

3. Bauer, B. *et al.* The ALPS project release 2.0: open source software for strongly correlated systems. *J. Stat. Mech.: Theory and Experiment* **2011**, P05001 (2011).
